# Quantum Plasmonic Dynamics Modeled via a Modified Langevin Noise Formalism: Numerical Studies of Single‐Photon Emission and Two‐Photon Interference

**DOI:** 10.1002/nap2.70236

**Published:** 2026-08-19

**Authors:** Jisang Seo, Hyunwoo Choi, Thomas E. Roth, Jie Zhu, Weng Cho Chew, Dong‐Yeop Na

**Affiliations:** ^1^ Department of Electrical Engineering Pohang University of Science and Technology Pohang Republic of Korea; ^2^ Elmore Family School of Electrical and Computer Engineering Purdue University West Lafayette Indiana USA

**Keywords:** beam shaping, bull's‐eye antenna, dissipative quantum electrodynamics, modified Langevin noise formalism, non‐Markovian, plasmonic Hong‐Ou‐Mandel effect, quantum plasmonics, spontaneous emission, surface plasmon polaritons, two‐photon interference

## Abstract

Recent studies have established and rigorously validated a modified Langevin noise formalism that enables macroscopic‐QED‐based quantization of electromagnetic fields in open and dissipative environments. Building on this foundation, a multimode Jaynes–Cummings framework has been developed and verified, providing an accurate description of atom–field interactions in lossy and radiative systems. In this work, we explore the potential of this formalism for nanophotonic applications by modeling representative quantum–plasmonic dynamics. In particular, we present detailed numerical examples for (i) two‐photon interference mediated by a quantum plasmonic beam splitter, and (ii) non‐Markovian dynamics of an atom located in plasmonic antennas and corresponding directional control of out‐coupled single‐photon fields. These results illustrate how the proposed modeling approach can provide quantitative insight into the behavior of plasmonic single‐photon sources and beam‐splitting structures.

## Graphical abstract

Based on the modified Langevin noise formalism and BA‐MA theory, optimized computational formulations are demonstrated for quantum plasmonic simulations without explicit BA–MA mode construction. Plasmonic Hong‐Ou‐Mandel interference, g2, atomic population, and single‐photon probability amplitudes are efficiently analyzed, revealing directional single‐photon emission from plasmonic nanoantennas in realistic lossy environments.

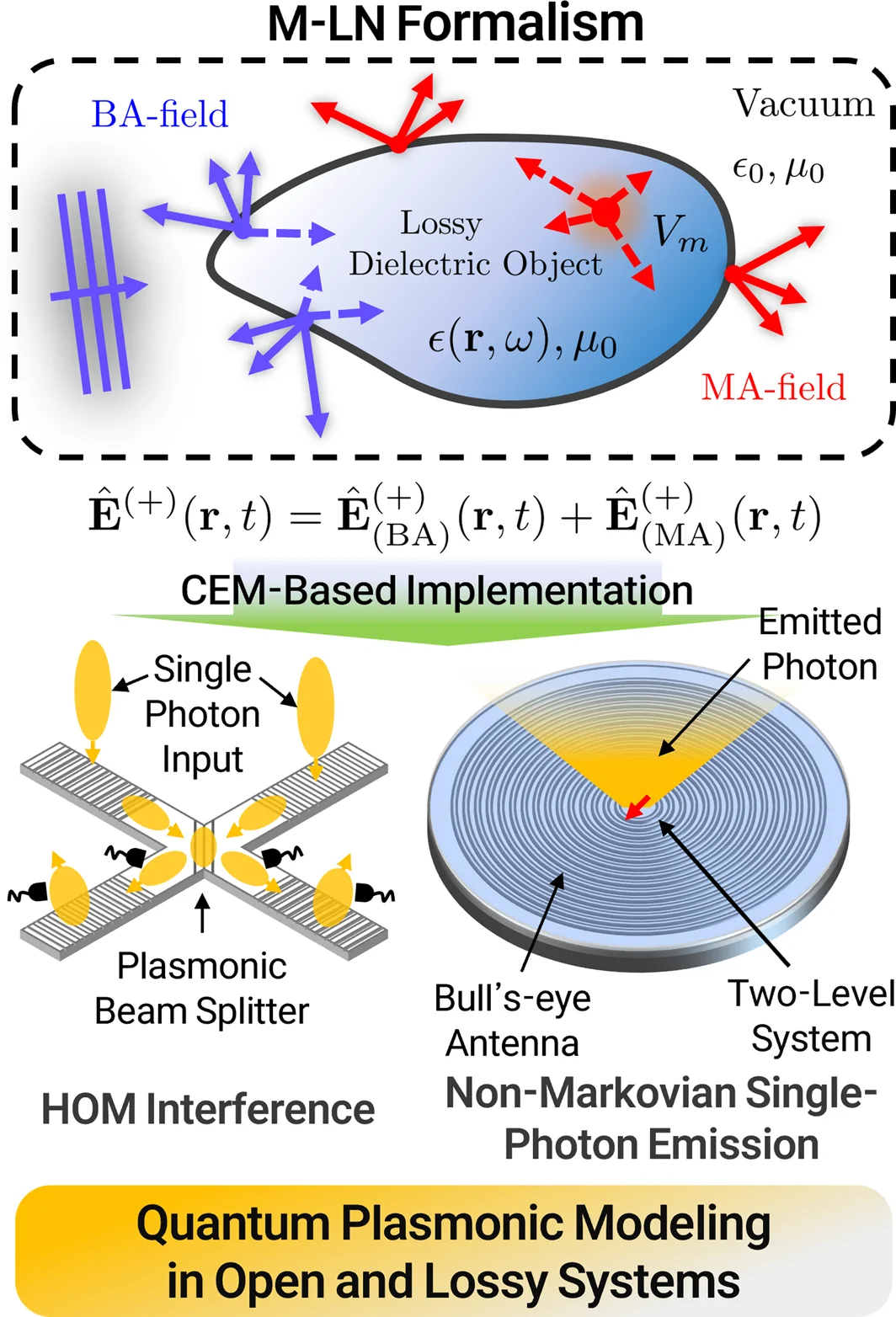

## 1 Introduction

Practical quantum technologies—ranging from quantum communication to quantum sensing and on‐chip quantum information processing—operate almost exclusively in open and dissipative electromagnetic (EM) environments [1, 2, 3, 4]. Unlike idealized cavity quantum electrodynamics (QED) or lossless waveguide settings, realistic photonic platforms inevitably exhibit absorption, radiation leakage, and complex modal interactions with their surrounding media. These effects fundamentally shape the dynamics of quantum states, influence coherence times, and determine the performance limits of integrated quantum systems.

Among various platforms, plasmonics‐based quantum information science and technology (QIST) has emerged as a particularly compelling direction [5, 6, 7, 8, 9]. Surface plasmon polaritons (SPPs) enable subwavelength confinement, ultrafast field localization, and strong enhancement of light–matter interactions, offering routes toward compact quantum circuits, ultrabright single‐photon sources, and chip‐scale quantum interference devices. A representative example is the Hong–Ou–Mandel (HOM) effect, a powerful probe of photon indistinguishability in quantum optics [10]. When two indistinguishable photons enter a 50:50 beam splitter simultaneously, the second‐order correlation of the output ideally vanishes, revealing nonclassical two‐photon interference. Recent experiments [6, 11, 12] have demonstrated that indistinguishable bosonic excitations in SPP channels also exhibit HOM interference through plasmonic beam splitting. Because conventional quantum–optical components (e.g., beam splitters and phase shifters) remain bulky, the preservation of bosonic interference in plasmonic media underscores their promise as a compact alternative for integrated quantum photonic platforms. Beyond two‐photon interference, plasmonic structures also enable ultrafast control, beam shaping, and directional steering of atomic spontaneous emission, as demonstrated using nanocube antennas [13] and bull's‐eye antennas [14, 15 ]. Such capabilities highlight the potential of plasmonic platforms to serve as highly efficient and tunable single‐photon sources.

However, the same metallic and nanostructured environments that provide strong confinement also introduce substantial Ohmic and radiative losses. Plasmonic materials such as gold, silver, and hybrid metasurfaces possess dispersion profiles that satisfy the Kramers–Kronig relations, implying that dispersion and intrinsic Ohmic loss inevitably coexist. As a result, plasmonic structures become non‐Hermitian: their eigenmodes lose orthonormality and their eigenfrequencies acquire imaginary parts, preventing the direct application of standard harmonic‐oscillator second quantization based on Hermitian‐mode diagonalization in terms of canonical creation and annihilation operators [16, 17, 18, 19].

To describe these loss mechanisms, Lindblad‐based approaches have represented the electromagnetic dynamics through selected quasinormal modes (QNMs), polariton modes, or other effective subsystems, while treating residual reservoirs through loss and transition rates under Born–Markov assumptions [20, 21, 22]. Moreover, beyond such Born–Markov approximations, environmental memory can be incorporated into reduced‐state dynamics using methods such as free‐pole hierarchical equations of motion (FP‐HEOM), which represent bath correlations through a hierarchy of auxiliary density operators [23, 24, 25]. Such reduced‐state formulations offer compact descriptions when selected system variables are sufficient.

Complementary to these reduced‐state descriptions, the conventional Langevin noise (LN) formalism [26, 27] introduces medium‐assisted (MA) fields—fluctuating sources that restore correct commutation relations through the fluctuation–dissipation theorem (FDT). However, realistic nanophotonic systems also experience radiation loss due to open boundaries, which is not captured by MA fields alone [3, 28, 29]. Radiation leakage constitutes an additional non‐Hermitian channel and must be treated on equal footing with Ohmic dissipation. The modified Langevin noise (M–LN) formalism [1, 2, 3, 30, 31] resolves this limitation by introducing boundary‐assisted (BA) fields to compensate radiation loss, as illustrated in Figure 1.

**FIGURE 1:**
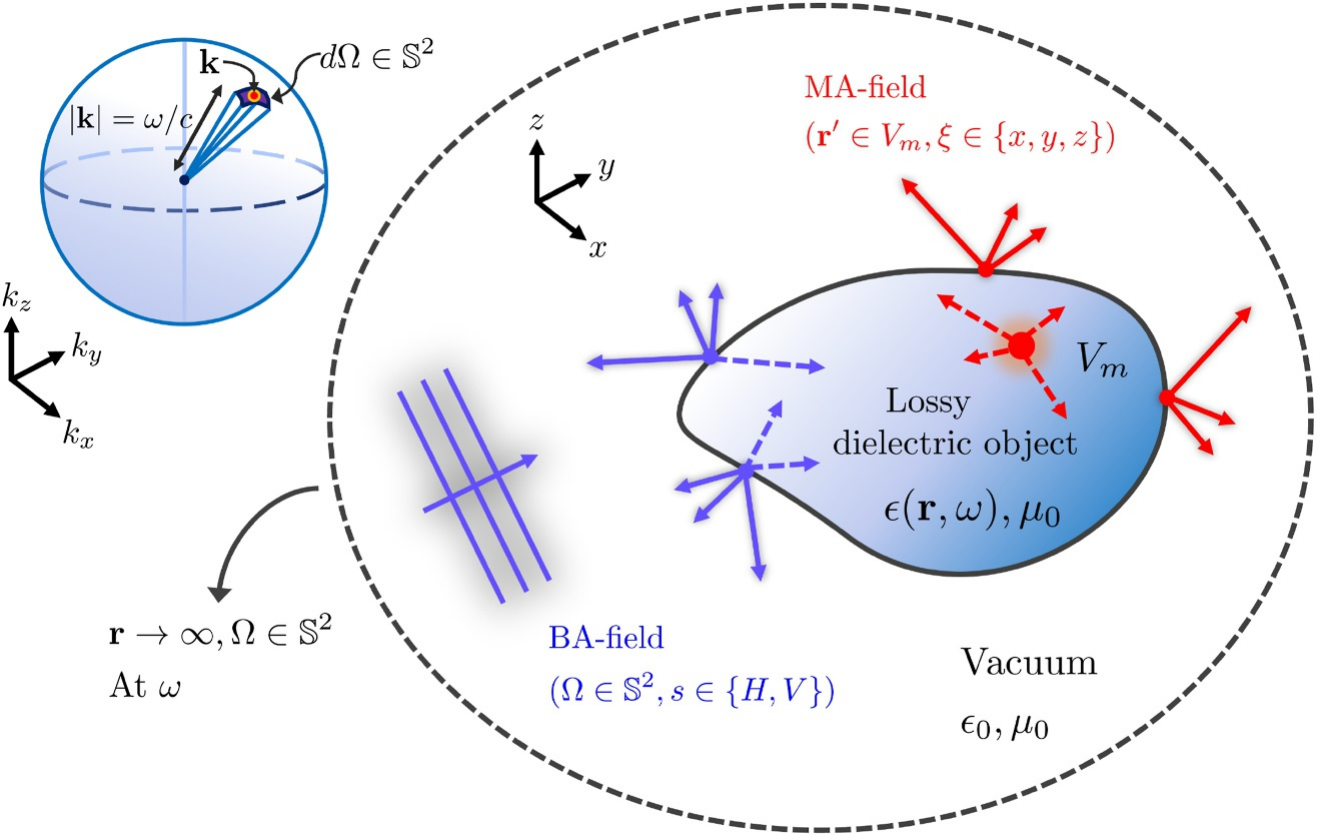
The modified Langevin noise formalism [1, 2, 3] employ two different fields for the quantization of open and dissipative EM systems: (i) Boundary‐assisted (BA) and (ii) medium‐assisted (MA) fields to balance the radiation and medium losses. Monochromatic BA and MA fields form the infinite degeneracy in terms of ($k\in S_{k}$, $s\in \left\{H,V\right\}$) and $\left(r^{\prime }\in V_{m},\xi \in \left\{x,y,z\right\}\right)$, respectively.

Building on this BA–MA quantization, the present formulation uses the sampled frequency‐dependent Maxwell Green tensor in both the emitter–field evolution and field reconstruction. This preserves geometry‐, dispersion‐, and propagation‐dependent electromagnetic information without first fitting the response to a few modes or effective rates, enabling finite propagation‐time effects and spatial single‐photon distributions to be evaluated within the same framework.

Several important successes of the M–LN formalism have been demonstrated [4, 30, 31, 32]. Among them, in [4], atoms were coupled explicitly to the BA–MA continuum to investigate non‐Markovian atomic dynamics in one‐dimensional settings, including dissipative cavity quantum electrodynamics, superradiance and subradiance, and entanglement sudden death. However, extending such explicit atom–continuum coupling strategy to higher‐dimensional systems is challenging, as the number of BA–MA continuum modes grows rapidly and becomes computationally intractable compared to the manageable sampling required in the one‐dimensional case. To address this obstacle, recent work introduced a computationally efficient approach for treating non‐Markovian spontaneous emission dynamics in open [33] and dissipative EM environments [34]. These studies primarily focused on simple open and dissipative cavity‐QED scenarios.

In this work, we leverage the M–LN formalism to two representative quantum–plasmonic problems. First, within the Heisenberg picture, we analyze two‐photon interference in a plasmonic beam‐splitting platform. Two initially separable single photons are incident from free space onto the input grating couplers, and HOM‐type interference is evaluated from the second‐order correlations of the out‐coupled free‐space and surface‐plasmon–polariton fields.

Notably, we show that the resulting expressions for the second‐order correlation and the single‐photon detection probability can be computed from classical EM simulations corresponding to the two incident single‐photon wavepackets. Second, within the Schrödinger picture, we investigate single‐photon spontaneous emission in a plasmonic nano‐antenna environment. By combining M–LN quantization with a multimode Jaynes–Cummings (MMJC) model, we derive a modified MMJC formulation that remains computationally efficient while capturing atom–plasmon–radiation coupling.

The key idea is to lump out the continuum degeneracy degrees of freedom (DoFs) and define effective DoFs that depend only on frequency, which are then coupled to the TLS.

## 2 Modified Langevin Noise Formalism

### 2.1 Theoretical Background

Consider a dispersive and lossy dielectric object occupying a volume $V_{m}$ in free space, whose permittivity is given by

(1) $$\begin{array}{ll} \epsilon \left(r,\omega \right) & =\epsilon_{0}\epsilon_{r}\left(r,\omega \right)=\epsilon_{0}\left[1+\chi_{e}\left(r,\omega \right)\right], \end{array}$$

where $\chi_{e}\left(r,\omega \right)$ is the electric susceptibility satisfying the Kramers–Kronig relations required for causality. For completeness, we note that the dielectric response of metallic nanostructures can, in general, be spatially nonlocal. Such corrections may originate from the nonlocal response of delocalized valence electrons [35], whereas tunneling‐induced charge transfer can become relevant across extremely narrow metallic gaps [36, 37]. These effects may require explicit modeling for nanometer‐scale features or subnanometer‐to‐few‐nanometer gaps (typically ∼ 1 nm or below [35, 36, 37]). In the present work, however, the plasmonic couplers and antennas under consideration have characteristic dimensions and gap sizes of several tens to hundreds of nanometers, or larger. We therefore employ a local dielectric response model, and a detailed treatment of nonlocal and tunneling corrections is left outside the scope of the present work.

Within the M–LN formalism, the transverse part of the EM field in such an open and dissipative environment is represented by the linear superposition of BA and MA fields. The positive‐frequency part of the electric field operator is expanded as

(2) $$\begin{array}{ll} {\hat{E}}^{\left(+\right)}\left(r,t\right) & ={\hat{E}}_{\left(\text{BA}\right)}^{\left(+\right)}\left(r,t\right)+{\hat{E}}_{\left(\text{MA}\right)}^{\left(+\right)}\left(r,t\right), \end{array}$$

where the BA‐field contribution takes the form

(3) $$\begin{array}{ll} {\hat{E}}_{\left(\text{BA}\right)}^{\left(+\right)}\left(r,t\right) & =i\int_{0}^{\infty }d\omega \int_{S^{2}}k^{2}d\Omega \sum_{s\in \left\{H,V\right\}}E_{B}\left(r;\Omega ,s,\omega \right)\;{\hat{a}}_{B}\left(\Omega ,s,\omega \right)e^{-i\omega t}, \end{array}$$

and the MA‐field contribution is expressed as

(4) $$\begin{array}{ll} {\hat{E}}_{\left(\text{MA}\right)}^{\left(+\right)}\left(r,t\right) & =i\int_{0}^{\infty }d\omega \int_{V_{m}}dr^{\prime }\sum_{\xi \in \left\{x,y,z\right\}}E_{M}\left(r;r^{\prime },\xi ,\omega \right)\;{\hat{a}}_{M}\left(r^{\prime },\xi ,\omega \right)e^{-i\omega t}. \end{array}$$

here, ${\hat{a}}_{B}$ and ${\hat{a}}_{M}$ denote annihilation operators associated with BA and MA fields, respectively; Ω is the solid angle on the unit sphere $S^{2}$; s denotes the plane‐wave polarization; ξ represents the dipole orientation within the lossy medium; and $E_{B}$ and $E_{M}$ correspond to the BA and MA field mode functions [1, 2, 3]. The mode functions entering the expansion are given by

(5) $$E_{B}\left(r;\Omega ,s,\omega \right)=\frac{1}{{\left(2\pi \right)}^{d/2}}\Phi_{\text{tot}}\left(r;\Omega ,s,\omega \right)\sqrt{\frac{\hbar \omega }{2}},$$

(6) $$E_{M}\left(r;r^{\prime },\xi ,\omega \right)=k^{2}{\bar{G}}_{E}\left(r;r^{\prime },\omega \right)\cdot \hat{\xi }\sqrt{\frac{\hbar \;Im\left[\epsilon \left(r^{\prime },\omega \right)\right]}{\pi \epsilon_{0}}},$$

where d denotes dimension, and k=ω/c is the free‐space wavenumber, $\Phi_{\text{tot}}$ is the total field resulting from the incident plane wave and its scattered field, and ${\bar{G}}_{E}$ denotes the dyadic Green's function for the electric field.

The BA–MA field modes possess the following important properties:

- BA fields originate from radiation fluctuations in free space. Each continuum degeneracy corresponds to an incident plane wave arriving from the infinitely extended boundary. Thus, a single degenerate BA mode is composed of an incident plane wave together with the associated scattered fields generated by dielectric objects. Consequently, BA fields exhibit degeneracy with respect to the plane‐wave propagation direction (solid angle Ω in k‐space) and polarization s.
- MA fields arise from fluctuations associated with medium Ohmic loss. Each continuum degeneracy is determined by the position and orientation of a noise point‐current source residing inside the absorbing dielectric region $V_{m}$. Accordingly, a single degenerate MA mode corresponds to the EM field generated by such a point‐current source within the medium. Therefore, MA fields exhibit degeneracy with respect to the source location $\left(r^{\prime }\in V_{m}\right)$ and orientation ξ.
- BA and MA field modes do not interact with one another; their fluctuation channels are independent and mutually decoupled. Such orthogonality has been rigorously derived analytically in [32], and its validity has also been numerically demonstrated in [3]. Specifically, as demonstrated in [3], the sum of the decay rates calculated independently from the BA and MA modes in a lossy dielectric environment perfectly matches the total spontaneous emission rate obtained via conventional methodologies [28, 38]. This exact agreement confirms the absence of any cross‐modal overlap. Due to this property, their respective dynamics evolve entirely independently without any mutual mixing.
- The combined set of BA and MA field modes satisfies the transverse modal completeness relation in open and dissipative electromagnetic environments. A rigorous dyadic derivation of the corresponding Green‐function integral relation was given in Ref. [32]. Thus, the imaginary part of the dyadic Green function can be expanded in terms of the BA and MA modal contributions as follows:

(7) $$\begin{array}{ll} \frac{\hbar \omega^{2}\mu_{0}}{\pi }\text{Im}\left[{\bar{G}}_{E}\left(r;r^{\prime },\omega \right)\right] & =\sum_{s\in \left\{H,V\right\}}\int_{S^{2}}k^{2}d\Omega \;E_{B}\left(r;\Omega ,s,\omega \right)⊗E_{B}^{*}\left(r^{\prime };\Omega ,s,\omega \right)\\ & +\sum_{\zeta =\left\{x,y,z\right\}}\int_{V_{m}}dr^{\prime \prime }E_{M}\left(r;r^{\prime \prime },\zeta ,\omega \right)⊗E_{M}^{*}\left(r^{\prime };r^{\prime \prime },\zeta ,\omega \right). \end{array}$$

More specifically, each BA field mode contains the total field consisting of

(8) $$\begin{array}{ll} \Phi_{\text{tot}}\left(r;\Omega ,s,\omega \right) & =\Phi_{\text{inc}}\left(r;\Omega ,s,\omega \right)+\Phi_{\text{sca}}\left(r;\Omega ,s,\omega \right) \end{array}$$

where $\Phi_{\text{inc}}\left(r;\Omega ,s,\omega \right)={\hat{e}}_{s}e^{ik\cdot r}$ and the resulting scattered field can be found from a plane‐wave‐scattering problem stated by

(9) $$\nabla \times \nabla \times \Phi_{\text{sca}}\left(r;\Omega ,s,\omega \right)-\frac{\omega^{2}}{c^{2}}\epsilon_{r}\left(r,\omega \right)\Phi_{\text{sca}}\left(r;\Omega ,s,\omega \right)=\frac{\omega^{2}}{c^{2}}\chi_{e}\left(r,\omega \right)\Phi_{\text{inc}}\left(r;\Omega ,s,\omega \right).$$

It is important to note that this result does not imply that the BA modes neglect the effects of material loss. As shown in Equation (9), the BA modes are constructed by solving the electromagnetic scattering problem that explicitly includes the imaginary part of the permittivity (or electric susceptibility). Through this process, the photon loss induced by the material is naturally encoded within the BA modes.

On the other hand, each MA field mode corresponds to an EM field created by a point current source in dispersive and absorbing dielectric objects; hence, it has a dyadic Green's function governed by

(10) $$\nabla \times \nabla \times {\bar{G}}_{E}\left(r;r^{\prime },\omega \right)-\frac{\omega^{2}}{c^{2}}\epsilon_{r}\left(r,\omega \right){\bar{G}}_{E}\left(r;r^{\prime },\omega \right)=\bar{I}\delta \left(r-r^{\prime }\right).$$

Two different annihilation operators, that is, ${\hat{a}}_{B}\left(\Omega ,s,\omega \right)$ and ${\hat{a}}_{M}\left(r^{\prime },\xi ,\omega \right)$, are associated with BA and MA fields, respectively. These operators satisfy the standard bosonic commutation relations,

(11) $$\left[{\hat{a}}_{B}\left(\Omega ,s,\omega \right),{\hat{a}}_{B}^{†}\left(\Omega^{\prime },s^{\prime },\omega^{\prime }\right)\right]=\hat{I}\frac{1}{k^{2}}\delta \left(\Omega -\Omega^{\prime }\right)\delta_{s,s^{\prime }}\delta \left(\omega -\omega^{\prime }\right),$$

(12) $$\left[{\hat{a}}_{M}\left(r^{\prime },\xi ,\omega \right),{\hat{a}}_{M}^{†}\left(r_{m},\xi^{\prime },\omega^{\prime }\right)\right]=\hat{I}\delta \left(r^{\prime }-r_{m}\right)\delta_{\xi ,\xi^{\prime }}\delta \left(\omega -\omega^{\prime }\right),$$

(13) $$\left[{\hat{a}}_{M}\left(r^{\prime },\xi ,\omega \right),{\hat{a}}_{B}^{†}\left(\Omega ,s,\omega \right)\right]=0$$

and take multimode Fock states as eigenstates of their number operators.

The resulting field Hamiltonian operator can be written by the two creation and annihilation operators that diagonalize the Hamiltonian, as given in Eq. (4.37) of Ref. [1],

(14) $$\begin{array}{ll} \hat{H} & =\int_{0}^{\infty }d\omega \int_{S^{2}}k^{2}d\Omega \sum_{s\in \left\{H,V\right\}}\hbar \omega {\hat{a}}_{B}^{†}\left(\Omega ,s,\omega \right){\hat{a}}_{B}\left(\Omega ,s,\omega \right)+\int_{0}^{\infty }d\omega \int_{V_{m}}dr^{\prime }\sum_{\xi \in \left\{x,y,z\right\}}\hbar \omega {\hat{a}}_{M}^{†}\left(r^{\prime },\xi ,\omega \right){\hat{a}}_{M}\left(r^{\prime },\xi ,\omega \right). \end{array}$$

Replacing all the degeneracy indices (i.e., Ω, s, $r^{\prime }$, and ξ) with a single continuum degeneracy index λ, one can write the unified expression by

(15) $${\hat{E}}^{\left(+\right)}\left(r,t\right)=i\int_{0}^{\infty }d\omega \int_{D_{\lambda }}d\lambda \;E_{\omega ,\lambda }\left(r\right){\hat{a}}_{\omega ,\lambda }e^{-i\omega t}$$

with the field Hamiltonian

(16) $${\hat{H}}_{\text{field}}=\int_{0}^{\infty }d\omega \int_{D_{\lambda }}d\lambda \;\hbar \omega {\hat{a}}_{\omega ,\lambda }^{†}{\hat{a}}_{\omega ,\lambda }.$$

Finally, for the numerical evaluation of the BA modes and the dyadic Green tensor, a scattering boundary condition (SBC) and a perfectly matched layer (PML) are employed to impose the outgoing‐radiation condition of the unbounded exterior domain. These boundary treatments are purely numerical and do not represent physical material absorption. Accordingly, neither the SBC nor the PML is included in the physical lossy‐material domain $V_{m}$ or assigned additional MA DoFs.

## 3 Two‐Photon Interference

We consider a three‐dimensional simulation scenario in which two independent single photons are incident on a plasmonic structure from free space. Each photon is assumed to be both spatially and temporally localized and is modeled as a Gaussian wavepacket represented by a superposition of single‐photon Fock states over multiple EM field modes. It is important to emphasize that, since the incident single photons originate from free space, their mode expansion must be expressed exclusively in terms of BA field modes.

The MA DoFs may, in principle, be thermally populated at finite temperature, with the corresponding mean occupation number given by the Bose–Einstein distribution,

(17) $$n_{\text{th}}\left(\omega ,T\right)=\frac{1}{\exp\left(\hbar \omega /k_{B}T\right)-1},$$

(18) $$\langle {\hat{a}}_{M}^{†}\left(r,\xi ,\omega \right){\hat{a}}_{M}\left(r^{\prime },\xi^{\prime },\omega^{\prime }\right)\rangle =n_{\text{th}}\left(\omega ,T\right)\delta_{\xi \xi^{\prime }}\delta \left(r-r^{\prime }\right)\delta \left(\omega -\omega^{\prime }\right).$$

At λ=808nm and T=300K, $\hbar \omega /k_{B}T≃59$, yielding $n_{\text{th}}∼10^{-26}$. Accordingly, initial thermal excitations of the MA field modes are neglected.

### 3.1 Initial Quantum State

An initial quantum state for a single photon riding on a wavepacket (i.e., spatially and temporally localized) coming from infinity can be modeled as

(19) $$|\Psi^{\left(1\right)}\left(t_{0}\right)\rangle =\int_{0}^{\infty }d\omega \int_{S^{2}}k^{2}\;d\Omega \sum_{s\in \left\{H,V\right\}}\tilde{g}\left(\Omega ,s,\omega \right)\;|1_{B}\left(\Omega ,s,\omega \right)\rangle =\int_{0}^{\infty }d\omega \int_{S^{2}}k^{2}\;d\Omega \sum_{s\in \left\{H,V\right\}}\tilde{g}\left(\Omega ,s,\omega \right)\;{\hat{a}}_{B}^{†}\left(\Omega ,s,\omega \right)|0\rangle ,$$

where $\tilde{g}$ denotes the spectral probability amplitudes associated with the single‐photon Fock state $|1_{B}\rangle$ of the BA field modes. Due to the probability normalization condition, the spectral amplitude must satisfy

(20) $$\int_{0}^{\infty }d\omega \int_{S^{2}}k^{2}\;d\Omega \sum_{s\in \left\{H,V\right\}}{\left|\tilde{g}\left(\Omega ,s,\omega \right)\right|}^{2}=1.$$

The initial Gaussian function, which specifies the spatial profile of the single photon in three‐dimensional space, is assumed to be localized and is given by

(21) $$g_{0}\left(r\right)=\exp\left[-\frac{|{\left(r-r_{0}\right)}_{\|}{|}^{2}}{2\sigma_{\|}^{2}}-\frac{|{\left(r-r_{0}\right)}_{⊥}{|}^{2}}{2\sigma_{⊥}^{2}}\right]\exp\left[ik_{p}\cdot \left(r-r_{0}\right)\right],$$

(22) $${\left(r-r_{0}\right)}_{\|}=\left[{\hat{k}}_{p}\cdot \left(r-r_{0}\right)\right]{\hat{k}}_{p},\;{\left(r-r_{0}\right)}_{⊥}=\left(r-r_{0}\right)-{\left(r-r_{0}\right)}_{\|}.$$

Here, ${\hat{k}}_{p}=k_{p}/|k_{p}|$ specifies the central propagation direction of the Gaussian wavepacket. The vector $r_{0}$ denotes the initial center of the localized photon wavepacket, whereas $\sigma_{\|}$ and $\sigma_{⊥}$ characterize the longitudinal pulse length and the transverse spatial width, respectively. The phase factor $\exp\left[ik_{p}\cdot \left(r-r_{0}\right)\right]$ represents the carrier plane wave of the incident photon and assigns the wavepacket a central wavevector $k_{p}$. The corresponding spectral component is obtained through

(23) $${\tilde{g}}_{0}\left(k\right)=\int_{R^{3}}d^{3}rg_{0}\left(r\right)\;\exp\left(-ik\cdot r\right),$$

where in the three‐dimensional space $R^{3}$, a wavevector $k\left(\Omega ,\omega \right)=k\left(\omega \right)\hat{k}\left(\Omega \right)$ with $\hat{k}\left(\Omega \right)=\sin\;\theta \;\cos\;\phi \;\hat{x}+\sin\;\theta \;\sin\;\phi \;\hat{y}+\cos\;\theta \;\hat{z}$, Ω=(θ,ϕ), and the radial wavevector variable is determined by the frequency as k(ω)=ω/c for free‐space propagation. Using this localization spectrum, the spectral probability amplitude of a Gaussian wavepacket centered at $k_{p}$ can be written as

(24) $$\tilde{g}\left(\Omega ,s,\omega \right)=N\chi_{s}\;{\tilde{g}}_{0}\left[k\left(\Omega ,\omega \right)\right],$$

where N is a normalization factor chosen to satisfy the probability normalization condition in Equation (20), and $\chi_{s}$ denotes the polarization amplitude.

When the above spectral amplitude is taken in the plane‐wave basis (which corresponds to the BA field modes in the absence of scattering targets), the resulting quantum state models the free‐space propagation of the initial Gaussian wavepacket. In contrast, when the same spectral amplitude is expanded in the full BA field modes in the presence of scattering objects, the time evolution naturally describes a wavepacket incident from free space, interacting with the targets, and subsequently scattering back into the environment. Therefore, in order to model the plasmonic HOM effect—where two separable single photons, each riding on a Gaussian wavepacket, propagate from free space toward the plasmonic platform—we employ this strategy to construct the initial quantum states of the incident photons.

With this prescription for generating a single‐photon state, we can construct a two‐photon incident state. If the two photons are unentangled, the initial state must be separable and can therefore be written as the tensor product of the two single‐photon states:

(25) $$\begin{array}{ll} |\Psi^{\left(2\right)}\left(t_{0}\right)\rangle & =|\Psi_{\left(L\right)}^{\left(1\right)}\left(t_{0}\right)\rangle ⊗|\Psi_{\left(R\right)}^{\left(1\right)}\left(t_{0}\right)\rangle \\ & =\left[\int_{0}^{\infty }d\omega \int_{S^{2}}k^{2}\;d\Omega \sum_{s\in \left\{H,V\right\}}{\tilde{g}}_{\left(L\right)}\left(\Omega ,s,\omega \right)\;|1_{B}\left(\Omega ,s,\omega \right)\rangle \right]\\ & ⊗\left[\int_{0}^{\infty }d\omega \int_{S^{2}}k^{2}\;d\Omega \sum_{s\in \left\{H,V\right\}}{\tilde{g}}_{\left(R\right)}\left(\Omega ,s,\omega \right)\;|1_{B}\left(\Omega ,s,\omega \right)\rangle \right], \end{array}$$

where the subscripts L and R label the left‐ and right‐incident photons, respectively.

### 3.2 Second‐Order Correlation and Single‐Photon Detection Probability

We evaluate the polarization‐unresolved second‐order correlation function

$$g^{\left(2\right)}\left(r_{\alpha },t_{\alpha };r_{\beta },t_{\beta }\right)=\frac{\sum_{i,j}\langle A_{ij}|A_{ij}\rangle }{B_{\alpha }B_{\beta }},$$

where

(26) $$|A_{ij}\rangle ={\hat{E}}_{\left(BA\right),j}^{\left(+\right)}\left(r_{\beta },t_{\beta }\right){\hat{E}}_{\left(BA\right),i}^{\left(+\right)}\left(r_{\alpha },t_{\alpha }\right)|\Psi^{\left(2\right)}\left(t_{0}\right)\rangle ,$$

(27) $$B_{\zeta }=\langle \Psi^{\left(2\right)}\left(t_{0}\right)\left|{\hat{E}}_{\left(BA\right)}^{\left(-\right)}\left(r_{\zeta },t_{\zeta }\right)\cdot {\hat{E}}_{\left(BA\right)}^{\left(+\right)}\left(r_{\zeta },t_{\zeta }\right)\right|\Psi^{\left(2\right)}\left(t_{0}\right)\rangle ,$$

for i,j∈{x,y,z} and ζ=α,β.

Here, the second‐order correlation function is expressed solely using the BA field. As discussed in Section 2.1, the BA and MA sectors are dynamically decoupled due to their orthogonality. Because the MA sector remains in the vacuum state throughout the evolution, all normally ordered expectation values involving the MA field identically vanish:

(28) $$\langle {\hat{E}}_{\left(MA\right)}^{\left(-\right)}\cdot {\hat{E}}_{\left(MA\right)}^{\left(+\right)}\rangle =0,\;\langle {\hat{E}}_{\left(BA\right)}^{\left(-\right)}\cdot {\hat{E}}_{\left(MA\right)}^{\left(+\right)}\rangle =\langle {\hat{E}}_{\left(MA\right)}^{\left(-\right)}\cdot {\hat{E}}_{\left(BA\right)}^{\left(+\right)}\rangle =0.$$

Consequently, BA–MA cross contributions and pure MA contributions are eliminated, allowing $g^{\left(2\right)}$ to be completely determined by the BA contribution alone.

Using the orthonormality of Fock states and bosonic commutation relations, the state associated with each pair of detected Cartesian components becomes

(29) $$\begin{array}{ll} |A_{ij}\rangle & =\left[E_{j}^{\left(+\right)}\left(r_{\beta },t_{\beta };L\right)E_{i}^{\left(+\right)}\left(r_{\alpha },t_{\alpha };R\right)\right.\\ & +\left.E_{j}^{\left(+\right)}\left(r_{\beta },t_{\beta };R\right)E_{i}^{\left(+\right)}\left(r_{\alpha },t_{\alpha };L\right)\right]|0\rangle . \end{array}$$

Here, for ζ=α,β, $E^{\left(+\right)}\left(r_{\zeta },t_{\zeta };\eta \right)$ denotes the classical field envelope and can be expressed as

(30) $$E^{\left(+\right)}\left(r_{\zeta },t_{\zeta };\eta \right)≜\int_{0}^{\infty }d\omega \int_{S^{2}}k^{2}\;d\Omega \sum_{s\in \left\{H,V\right\}}{\tilde{g}}_{\left(\eta \right)}\left(\Omega ,s,\omega \right)\;E_{B}\left(r_{\zeta };\Omega ,s,\omega \right)\;e^{-i\omega t_{\zeta }},$$

and the common factor $i^{2}=-1$ has been omitted, as it does not affect $\langle A_{ij}|A_{ij}\rangle$.

Likewise, the single‐photon detection probability becomes

(31) $$\begin{array}{ll} \text{SPDP}\left(r_{\zeta },t_{\zeta }\right)=B_{\zeta } & ={\left|E^{\left(+\right)}\left(r_{\zeta },t_{\zeta };L\right)\right|}^{2}+{\left|E^{\left(+\right)}\left(r_{\zeta },t_{\zeta };R\right)\right|}^{2}. \end{array}$$

The physical meaning of $\sum_{i,j}\langle A_{ij}|A_{ij}\rangle$ is the coincidence‐detection probability at $\left(r_{\alpha },t_{\alpha }\right)$ and $\left(r_{\beta },t_{\beta }\right)$, whereas $B_{\alpha }$ (or $B_{\beta }$) describes the probability of detecting a single photon at $\left(r_{\alpha },t_{\alpha }\right)$ (or $\left(r_{\beta },t_{\beta }\right)$), respectively. It is interesting to observe that the resulting expressions involve products of field envelopes that can be evaluated using classical EM propagation; however, it is important to emphasize that these forms are obtained only after a quantum–optical evaluation of the field operators acting on the two‐photon state. In particular, the structure of the coincidence term and the specific manner in which independently propagated wavepackets are combined are dictated by the underlying quantum correlations and bosonic commutation relations. Thus, although the required field propagation can be carried out using classical EM simulations, their postprocessing—and hence the computation of the second‐order correlation function—remains fundamentally governed by quantum optical principles, rather than constituting a purely classical description.

### 3.3 Simulation Results of Plasmonic Hong–Ou–Mandel Effects

Based on the developed numerical framework, we perform three‐dimensional simulations to observe plasmonic HOM interference using a structure based on that reported in [6]. The simulation scenario is illustrated in Figure 2. This structure consists of a thin gold (Au) film deposited on a silica substrate, with two input grating couplers and two output grating couplers arranged on the left, right, top, and bottom sides of the structure. A Bragg‐reflector‐type plasmonic beam splitter composed of three protruding parallel ridges is positioned at the center. For the beam splitter, the center‐to‐center spacing between adjacent ridges and the in‐plane width of each ridge are set to 610nm and 150nm, respectively. All other geometrical parameters follow those of the structure reported in [6]. The refractive index of silica is set to n=1.45, and the dispersive permittivity ϵ(r,ω) of Au is modeled using experimental optical data from [39].

**FIGURE 2:**
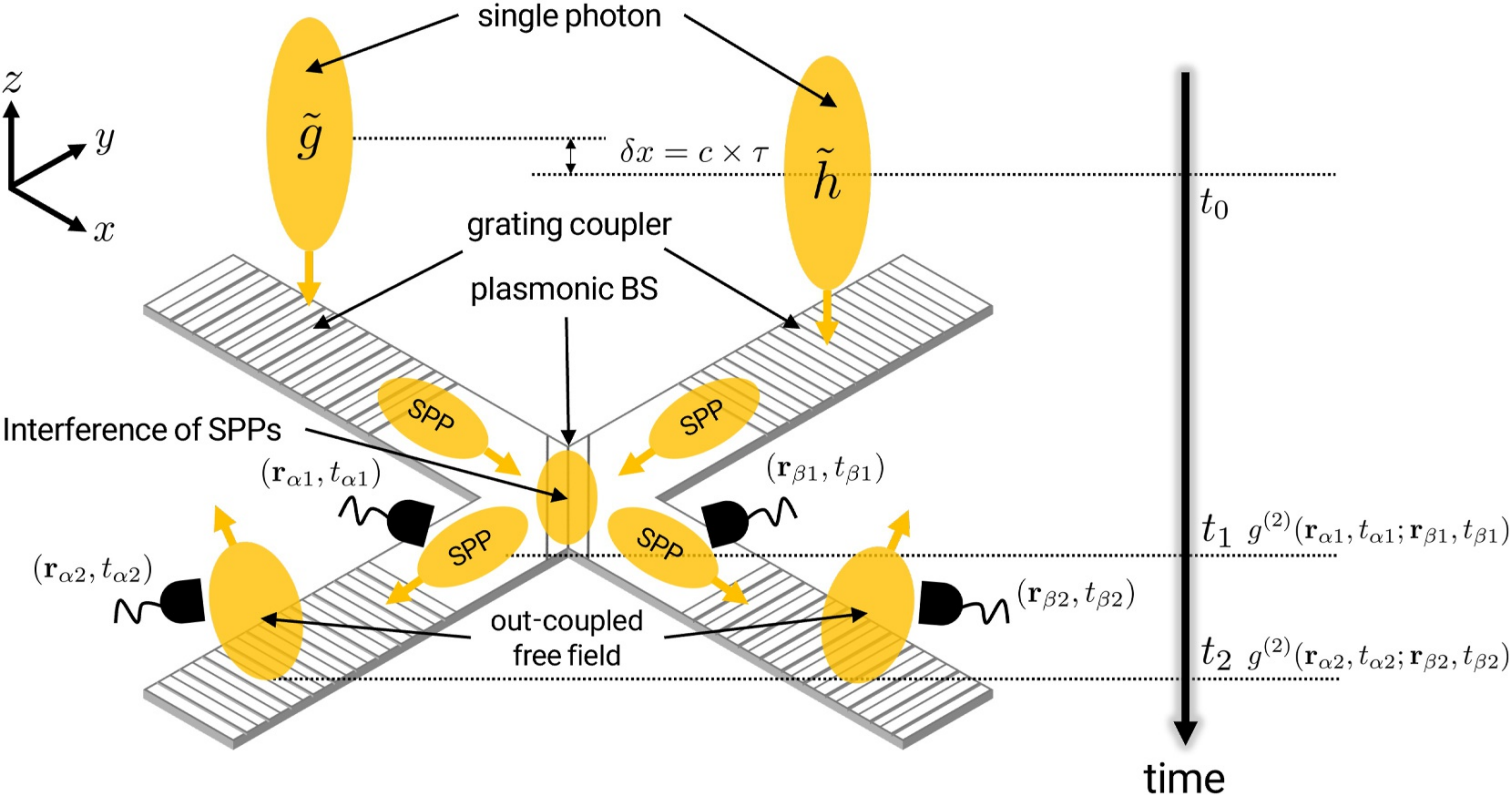
Numerical simulation scenario for observing the quantum plasmonic Hong–Ou–Mandel effect in 3D.

We consider two unentangled single photons in vacuum incident on the grating couplers. Each photon has a center wavelength of 808nm and a temporal full width at half maximum (FWHM) of 0.02ps. Because the two input ports are oriented orthogonally, the incident electric fields corresponding to the two ports are polarized along the x and y directions, respectively, so as to efficiently excite the associated SPP modes. The excited SPPs propagate toward the plasmonic BS, undergo plasmonic interference, and are subsequently converted back into out‐coupled photons by the grating couplers. The out‐coupled photons are then detected at two photodetectors (at time $t_{2}$ in Figure 2), thereby generating a second‐order correlation signal. In addition, we evaluate second‐order correlations directly from the SPP fields before their conversion into free‐space photons (at time $t_{1}$ in Figure 2) to verify that SPPs, being bosonic quasiparticles, can also exhibit HOM visibility.

Following the numerical framework developed in the previous section, we evaluate the plasmonic HOM interference as a function of the relative delay between the two input photons. The delay is introduced by temporally shifting one of the two input wavepackets, whose spectral amplitude is denoted by $\tilde{h}$. For each frequency component within the single‐photon bandwidth, a frequency‐domain FEM simulation is performed for an incident Gaussian beam. By exploiting the mirror symmetry about the beam splitter, only one input port is simulated directly, whereas the response for the other port is obtained through the corresponding symmetry operation. The time‐dependent wavepackets are then reconstructed by weighting and coherently superposing the frequency‐domain field solutions according to their Gaussian spectral distributions. Through this procedure, we obtain the positive‐frequency fields $E^{\left(+\right)}\left(r,t;\xi \right)$ for the two input channels, where ξ=L,R. All simulations were performed on a dual Intel Xeon Gold 6430 processor system with 1024 GB of RAM. The FEM simulations were carried out using COMSOL Multiphysics Wave Optics Module, whereas the quantum part of the simulations was performed using in‐house MATLAB code. Excluding visualization, the FEM simulations were conducted at 51 frequency points, requiring 42,375 s of computation time and reaching a peak memory footprint of 538.56 GB.

Figure 3 shows the evolution of the single‐photon detection probability in the presence of the BS. The two localized input photons are first converted into SPPs via the grating couplers, propagate toward the BS where plasmonic interference occurs, and then reconvert into out‐coupled photons.

**FIGURE 3:**
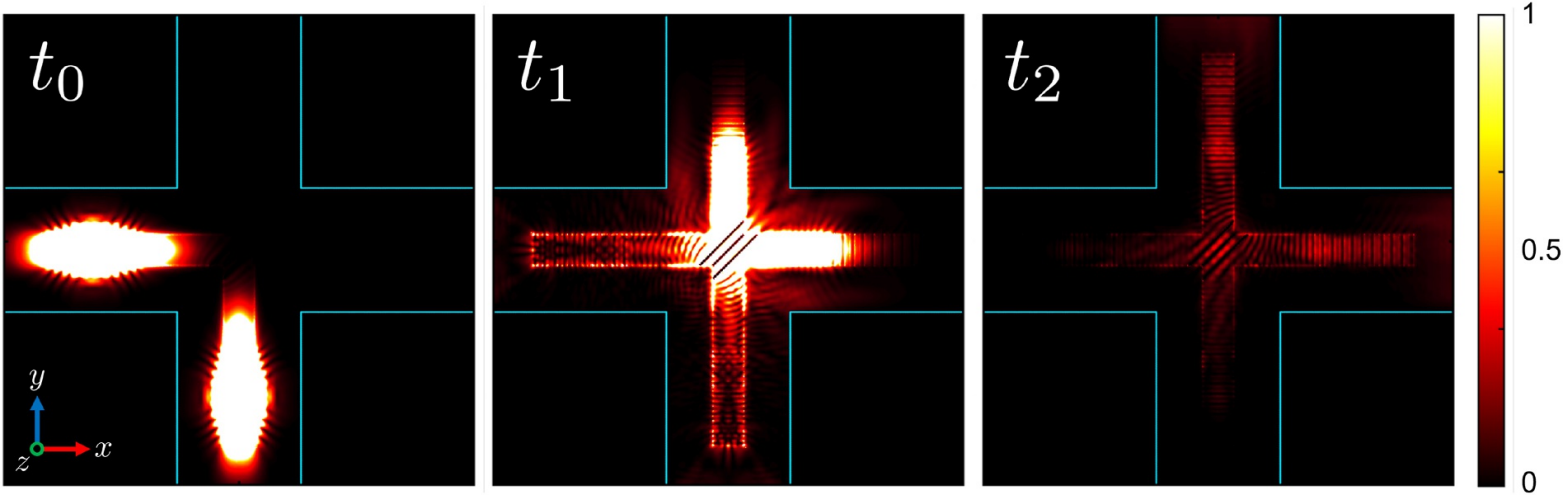
Single‐photon detection probability at successive time instants $t=t_{0},t_{1},t_{2}$ in the presence of the plasmonic beam splitter. The sequence shows photon‐to‐SPP conversion, SPP propagation and interference, and reconversion into out‐coupled photons. The color scale represents the relative single‐photon detection probability density in arbitrary units.

Figure 4 presents the computed normalized second‐order correlation $g^{\left(2\right)}\left(\tau \right)/g^{\left(2\right)}\left(\infty \right)$ as a function of the relative delay τ between the two input photons for three cases: (i) coincidence of out‐coupled free fields with the plasmonic BS present, (ii) coincidence of SPP fields with the BS present, and (iii) coincidence of out‐coupled fields in the absence of the BS. Cases (i) and (ii) clearly exhibit HOM dips for small τ (near the temporal FWHM of the photon wavepacket), whereas case (iii) displays almost unity correlation, as expected. The nonzero value at τ=0 arises from Ohmic loss in gold and the limited bandwidth over which the plasmonic BS achieves a true 50:50 splitting ratio.

**FIGURE 4:**
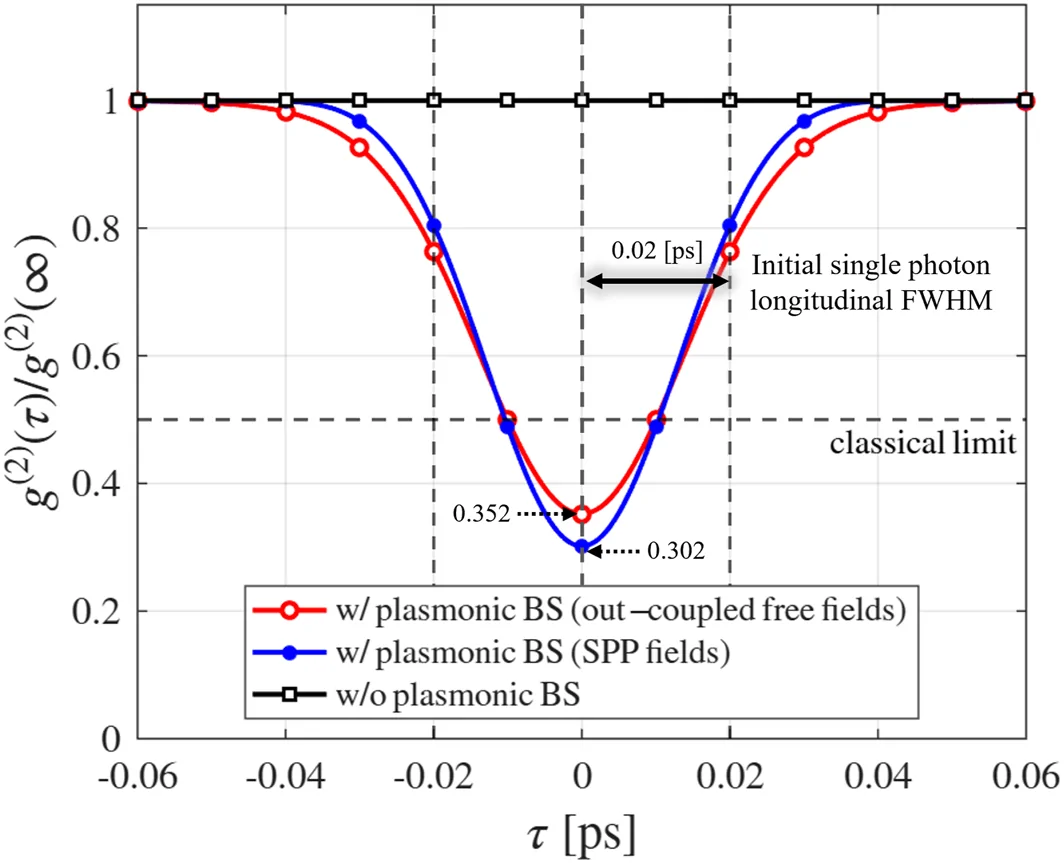
Second‐order correlation versus delay τ for three cases: (i) Plasmonic BS with coincidence of out‐coupled fields; (ii) plasmonic BS with coincidence of SPP fields; (iii) absence of the BS with coincidence of out‐coupled fields. At zero delay, the normalized correlations are $g_{\text{out}}^{\left(2\right)}\left(0\right)/g_{\text{out}}^{\left(2\right)}\left(\infty \right)=0.352$ and $g_{\text{SPP}}^{\left(2\right)}\left(0\right)/g_{\text{SPP}}^{\left(2\right)}\left(\infty \right)=0.302$.

It is noteworthy that case (ii) exhibits a slightly narrower HOM dip (larger effective quality factor) than case (i). This occurs because the out‐coupling process introduces additional dispersion and attenuation beyond those present in pure SPP propagation. Evaluating second‐order correlations from SPP‐field coincidences is possible only in numerical simulation, as direct measurement of bosonic statistics of SPPs remains extremely challenging experimentally.

## 4 Atom–Field Interaction

### 4.1 Multimode Jaynes–Cummings Model Combined With the M–LN Formalism

The multimode Jaynes–Cummings (MMJC) model generalizes atom–field interaction by including a continuum of EM field modes. It employs the two‐level system (TLS) approximation, in which the atom's full set of energy eigenstates is truncated to the ground and a single excited state that is nearly‐resonant with the relevant field modes. This approximation relies on the assumption that all other atomic transitions are sufficiently detuned so that their contributions to the dynamics can be neglected. By reducing the infinite‐dimensional atomic Hilbert space to an effective two‐dimensional subspace, the MMJC model provides a computationally efficient framework for describing spontaneous emission and light–matter coupling.

In the weak‐ and moderately strong‐coupling regimes, the system Hamiltonian under the rotating‐wave approximation (RWA) [40] takes the form

(32) $$\begin{array}{ll} \hat{H} & ={\hat{H}}_{\text{atom}}+{\hat{H}}_{\text{field}}+{\hat{H}}_{\text{interaction}}\\ & =\hbar \omega_{a}{\hat{\sigma }}_{+}{\hat{\sigma }}_{-}+\int_{0}^{\infty }d\omega \int_{D_{\lambda }}d\lambda \;\hbar \omega \;{\hat{a}}_{\omega ,\lambda }^{†}{\hat{a}}_{\omega ,\lambda }+\left[\int_{0}^{\infty }d\omega \int_{D_{\lambda }}d\lambda \;\hbar g_{\omega ,\lambda }\;{\hat{\sigma }}_{+}{\hat{a}}_{\omega ,\lambda }+H.c.\right], \end{array}$$

where $\omega_{a}$ denotes the TLS transition frequency, ${\hat{\sigma }}_{\pm }$ are the raising and lowering operators of the TLS, ${\hat{a}}_{\omega ,\lambda }$ annihilates a field quantum of frequency ω and mode index λ, and $g_{\omega ,\lambda }$ is the atom–field coupling coefficient,

(33) $$g_{\omega ,\lambda }=-\;i\;\frac{1}{\hbar }\;d_{a}\cdot E_{\omega ,\lambda }\left(r_{a}\right),$$

where $d_{a}$ is the atomic dipole moment and $E_{\omega ,\lambda }\left(r_{a}\right)$ is the mode function evaluated at the TLS position.

Since our primary interest lies in spontaneous emission from the TLS, we restrict the Hilbert space to the single‐excitation manifold and consider a general quantum state of the form

(34) $$|\psi \left(t\right)\rangle =C\left(t\right)|e,\left\{0\right\}\rangle +\int_{0}^{\infty }d\omega \int_{D_{\lambda }}d\lambda \;D_{\omega ,\lambda }\left(t\right)|g,1_{\omega ,\lambda }\rangle ,$$

where C(t) and $D_{\omega ,\lambda }\left(t\right)$ are the probability amplitudes for the TLS excitation and the single‐quantum field excitation, respectively.

The time evolution of this state is governed by the Schrödinger equation,

(35) $$i\hbar \;\frac{d}{dt}|\psi \left(t\right)\rangle =\hat{H}|\psi \left(t\right)\rangle .$$

Substituting Equations (32) and (34) into Equation (35) leads to two coupled equations for the probability amplitudes:

(36) $$i\frac{d}{dt}C\left(t\right)=\omega_{a}C\left(t\right)+\int_{0}^{\infty }d\omega \int_{D_{\lambda }}d\lambda \;g_{\omega ,\lambda }\;D_{\omega ,\lambda }\left(t\right),$$

(37) $$i\frac{d}{dt}D_{\omega ,\lambda }\left(t\right)=\omega \;D_{\omega ,\lambda }\left(t\right)+g_{\omega ,\lambda }^{*}\;C\left(t\right).$$

These equations describe the exchange of a single quantum between the TLS and the continuum of field modes. Also, this framework can simulate arbitrary single quanta initial states by setting values of C(0) and $D_{\omega ,\lambda }\left(0\right)$ as initial conditions for solving these coupled differential equations.

### 4.2 Computationally Efficient Approach

Directly solving Equation (37) requires knowledge of the entire continuum of BA–MA field modes. For numerical implementation, these continuum modes must be sampled appropriately, and such sampling becomes increasingly nontrivial in higher‐dimensional geometries, in contrast to the tractable one‐dimensional case [4]. In [34], a computationally efficient approach was developed by expressing the dynamics in terms of a memory kernel linked to the imaginary part of the dyadic Green's function. This formulation enables the dynamics to be computed using standard CEM solvers—such as auxiliary differential equation finite‐difference time‐domain with a total‐field/scattered‐field formulation (ADE‐FDTD‐TF/SF) or the FEM—without explicitly constructing the BA–MA modal continuum. Here, we propose an alternative computational strategy for TLS dynamics within the M–LN framework.

Multiplying Equation (37) by $g_{\omega ,\lambda }$ and integrating over λ yield

(38) $$\begin{array}{ll} i\frac{d}{dt}\int_{D_{\lambda }}d\lambda \;g_{\omega ,\lambda }D_{\omega ,\lambda }\left(t\right) & =\omega \int_{D_{\lambda }}d\lambda \;g_{\omega ,\lambda }D_{\omega ,\lambda }\left(t\right)+C\left(t\right)\int_{D_{\lambda }}d\lambda \;|g_{\omega ,\lambda }{|}^{2}. \end{array}$$

We now define

(39) $$E_{\omega }\left(t\right)≜\int_{D_{\lambda }}d\lambda \;g_{\omega ,\lambda }D_{\omega ,\lambda }\left(t\right),$$

The introduction of $E_{\omega }\left(t\right)$ reduces the number of DoFs in the BA–MA space from approximately $N_{\omega }N_{\lambda }$ to $N_{\omega }$, thereby transforming an otherwise computationally prohibitive dynamical problem into a tractable one.

Equation (39) allows Equations (36) and (37) to be rewritten as

(40) $$i\frac{d}{dt}C\left(t\right)=\omega_{a}C\left(t\right)+\int_{0}^{\infty }d\omega \;E_{\omega }\left(t\right),$$

(41) $$i\frac{d}{dt}E_{\omega }\left(t\right)=\omega \;E_{\omega }\left(t\right)+\Gamma \left(\omega \right)\;C\left(t\right),$$

where the coefficient

(42) Γ(ω)=∫Dλdλ|gω,λ|2=1ℏ2∫Dλdλ|da⋅Eω,λra|2=ω2μ0πℏd⋅ImG‾Era,ra;ω⋅d∗,

is obtained by applying the transverse modal completeness relation of the BA–MA field modes in Equation (7). In the memory‐kernel formulation [34], the field DoFs are integrated out, resulting in a time‐nonlocal equation for the atomic amplitude. In contrast, the present formulation retains the frequency‐resolved variables $E_{\omega }\left(t\right)$ and evolves them together with the atomic amplitude through a coupled first‐order dynamical system.

The coefficient Γ(ω) in Equation (42) represents the coupling‐strength density after integration over the BA–MA degeneracy DoFs. Accordingly, we define a band‐integrated effective coupling scale as

(43) $$g_{B}\equiv {\left[\int_{B}d\omega \;\Gamma \left(\omega \right)\right]}^{1/2},$$

and introduce the dimensionless ratio

(44) $$\eta_{B}\equiv \frac{g_{B}}{\omega_{a}}=\frac{1}{\omega_{a}}{\left[\sum_{n=1}^{N_{\omega }}\Gamma \left(\omega_{n}\right)\Delta \omega \right]}^{1/2}$$

for uniformly sampled frequencies over the simulation bandwidth B. In all cases considered, $\eta_{B}\ll 1$, providing an *a posteriori* consistency check supporting the use of the RWA in the present parameter range. We note that $\eta_{B}$ is an aggregate continuum‐coupling measure, rather than a unique single‐mode coupling constant, and therefore does not constitute a rigorous non‐RWA validation.

Once the dyadic Green's function is computed over the frequency bandwidth of interest and the frequencies are uniformly sampled with increment Δω, the modified coupled Equations (40) and (41) can be cast into a sparse linear dynamical system:

(45) $$i\frac{d}{dt}X\left(t\right)=\bar{M}\cdot X\left(t\right),$$

where

(46) $$X\left(t\right)={\left[C\left(t\right),\;\sqrt{\Delta \omega }\;E_{\omega_{1}}\left(t\right),\;\sqrt{\Delta \omega }\;E_{\omega_{2}}\left(t\right),\;\text{…},\;\sqrt{\Delta \omega }\;E_{\omega_{N_{\omega }}}\left(t\right)\right]}^{T},$$

and the nonzero entries of the evolution matrix $\bar{M}$ are

(47) $$\begin{array}{ll} {\left[\bar{M}\right]}_{1,1} & =\omega_{a},\\ {\left[\bar{M}\right]}_{i,i} & =\omega_{n},\;n=1,\text{…},N_{\omega },\;\;i=n+1,\\ {\left[\bar{M}\right]}_{1,i} & =\sqrt{\Delta \omega },\;i=2,\text{…},N_{\omega }+1,\\ {\left[\bar{M}\right]}_{i,1} & =\Gamma \left(\omega_{n}\right)\sqrt{\Delta \omega },\;i=2,\text{…},N_{\omega }+1,\\ {\left[\bar{M}\right]}_{i,j} & =0,\;\text{otherwise}. \end{array}$$

The system Equation (45) can be integrated numerically using either a fourth‐order Runge–Kutta method or the Crank–Nicolson scheme. To simulate the time evolution of Equation (46), the initial conditions for both C(t) and $E_{\omega }\left(t\right)$ must be specified. However, for an arbitrary initial state, the modal completeness relations cannot be utilized to calculate $E_{\omega }\left(t\right)$. Instead, we need to calculate all possible BA–MA modes and explicitly integrate over the entire degenerate space using Equation (39) to calculate $E_{\omega }\left(0\right)$. Although the framework remains theoretically valid for such general cases, calculating all degenerate modes is computationally expensive. Therefore, in this study, we assumed that only the emitter is initially excited, setting C(0)=1 and $D_{\omega ,\lambda }\left(0\right)=0$, which consequently yields $E_{\omega }\left(0\right)=0$.

It is important to emphasize that the original quantum state is normalized. When the continuum variables (ω,λ) are discretized with increments Δω and Δλ, the state vector in the original formulation is

(48) $$X_{\text{orig}}\left(t\right)≜{\left[C\left(t\right),\left\{\sqrt{\Delta \omega \;\Delta \lambda }\;D_{\omega ,\lambda }\left(t\right)\right\}\right]}^{T},$$

and it must satisfy the normalization condition

(49) $$X_{\text{orig}}^{†}\cdot X_{\text{orig}}\approx {\left|C\left(t\right)\right|}^{2}+\int_{0}^{\infty }d\omega \int_{D_{\lambda }}d\lambda {\left|D_{\omega ,\lambda }\left(t\right)\right|}^{2}=1.$$

However, the modified representation obeys

(50) $$\begin{array}{ll} X^{†}\left(t\right)\cdot X\left(t\right) & =|C\left(t\right){|}^{2}+\sum_{n=1}^{N_{\omega }}|E_{\omega_{n}}\left(t\right){|}^{2}\;\Delta \omega \\ & \approx |C\left(t\right){|}^{2}+\int_{0}^{\infty }d\omega \;|E_{\omega }\left(t\right){|}^{2}=|C\left(t\right){|}^{2}+\int_{0}^{\infty }d\omega \;{\left|\int_{D_{\lambda }}d\lambda \;g_{\omega ,\lambda }D_{\omega ,\lambda }\left(t\right)\right|}^{2}\neq 1. \end{array}$$

Thus, the evolution matrix $\bar{M}$ is generally nonunitary (and non‐Hermitian). Importantly, this nonunitarity does not indicate any loss of physical accuracy, nor does it represent physical dissipation or decoherence. Instead, it arises solely from the reduced representation introduced for computational efficiency, in which the continuum field DoFs (ω,λ) are projected onto the collective variables $E_{\omega }\left(t\right)$. As a consequence, the norm of the reduced state vector X(t) no longer corresponds to the total quantum probability of the full atom–field system. The underlying quantum dynamics governed by the original Hamiltonian remains strictly unitary, and all physical observables of interest—such as atomic populations and correlation functions—are computed from properly normalized quantities. Therefore, the apparent nonunitarity should be understood as a representation‐level artifact of the model reduction, rather than a physical or numerical inconsistency.

### 4.3 Single‐Photon Amplitude

In addition to the TLS dynamics, one may also wish to analyze the out‐coupled field or the radiated field generated by the TLS. A natural question then arises: after replacing $D_{\omega ,\lambda }\left(t\right)$ with the reduced quantity $E_{\omega }\left(t\right)$ for computational efficiency, is it still possible to compute the single‐photon amplitude or radiation characteristics? Otherwise, such a reduction would render the algorithm ineffective for field‐based analyses.

The key point is that the field behavior can indeed be reconstructed from $E_{\omega }\left(t\right)$. In fact, the quantity $E_{\omega }\left(t\right)$ contains exactly the information required to evaluate the single‐photon amplitude at any observation point. The underlying reason is that $E_{\omega }\left(t\right)$ is obtained by integrating $g_{\omega ,\lambda }D_{\omega ,\lambda }\left(t\right)$ over the degeneracy index λ, which is the same combination that enters the modal expansion of the electric field operator. We now explain this in detail below.

The single‐photon amplitude is defined by

(51) $$\begin{array}{ll} E_{\text{spa}}\left(r,t\right) & =\langle 0|\hat{E}\left(r,0\right)|\psi \left(t\right)\rangle =\int_{0}^{\infty }d\omega \int_{D_{\lambda }}d\lambda \;D_{\omega ,\lambda }\left(t\right)\;E_{\omega ,\lambda }\left(r\right), \end{array}$$

from which the first‐order correlation (or single‐photon detection probability) in the Schrödinger picture follows immediately as

(52) $$\text{SPDP}(r,t)=\left\langle \psi (t)|{\hat{E}}^{\left(-\right)}(r,0)\cdot {\hat{E}}^{\left(+\right)}(r,0)|\psi (t)\right\rangle ={\left|E_{\text{spa}}(r,t)\right|}^{2}.$$

Assuming that no photons are initially present, the formal solution of Equation (37) is

(53) $$D_{\omega ,\lambda }\left(t\right)=-ig_{\omega ,\lambda }^{*}\int_{0}^{t}dt^{\prime }\;C\left(t^{\prime }\right)\;e^{-i\omega \left(t-t^{\prime }\right)}.$$

Substituting Equation (53) into Equation (51), the single‐photon amplitude can be expressed explicitly in terms of the excited‐state amplitude C(t):

(54) $$\begin{array}{ll} E_{\text{spa}}\left(r,t\right) & =-i\int_{0}^{\infty }d\omega \int_{D_{\lambda }}d\lambda \;\left[\int_{0}^{t}dt^{\prime }\;C\left(t^{\prime }\right)e^{-i\omega \left(t-t^{\prime }\right)}\right]g_{\omega ,\lambda }^{*}E_{\omega ,\lambda }\left(r\right). \end{array}$$

This expression shows that the entire emitted field is generated solely by the temporal evolution of the atomic amplitude C(t). Next, using Equation (53) in the definition of $E_{\omega }\left(t\right)$, we obtain

(55) $$E_{\omega }\left(t\right)=\left[-i\int_{D_{\lambda }}d\lambda \;|g_{\omega ,\lambda }{|}^{2}\right]\int_{0}^{t}dt^{\prime }\;C\left(t^{\prime }\right)e^{-i\omega \left(t-t^{\prime }\right)}.$$

The λ‐integration over $|g_{\omega ,\lambda }{|}^{2}$ is evaluated through the BA–MA transverse modal completeness relation in (42). Substituting (42) into (55) gives the compact expression

(56) $$E_{\omega }\left(t\right)=-i\;\Gamma \left(\omega \right)\int_{0}^{t}dt^{\prime }\;C\left(t^{\prime }\right)e^{-i\omega \left(t-t^{\prime }\right)}.$$

Finally, inserting Equation (56) into Equation (54) and applying the modal completeness relation once more for the spatial part yields the Green‐tensor representation of the emitted single‐photon field:

(57) Espa(r,t)=μ0π∫0∞dωω2ImG‾Er,ra;ω⋅da∗Eω(t)−iΓ(ω).

### 4.4 Simulation Results of Spontaneous Emission in Plasmonic Antennas

We performed numerical simulations for a TLS placed in the plasmonic nanoantenna shown in Figure 5. The geometrical parameters of the nanoantenna were adopted from Ref. [14]. The optical properties of silver (Ag) and polymethyl methacrylate (PMMA) were modeled using the material data reported in Refs. [39, 41], respectively. For each case, simulations were performed on the same hardware and software with the two‐photon interference example case. In the process, excluding visualization, the simulation was performed across 101 frequency points, taking 71,799 s with a peak memory usage of 398.36 GB. In addition, the nanoantenna geometry possesses mirror symmetry with respect to both the xz and yz planes. By exploiting these symmetries through the method of images, with perfect electric conductor (PEC) and perfect magnetic conductor (PMC) symmetry boundary conditions, the computational domain was reduced to one quarter of the full structure. For reference, we compared our numerical results with those obtained using the spectral function approach (SFA) [3]. Within the Markov approximation, the SFA estimates the spontaneous‐emission rate from the imaginary part of the dyadic Green's function evaluated at the TLS transition frequency. The resulting Markovian population decay therefore provides a useful baseline for assessing the non‐Markovian behavior captured by our time‐domain simulations.

**FIGURE 5:**
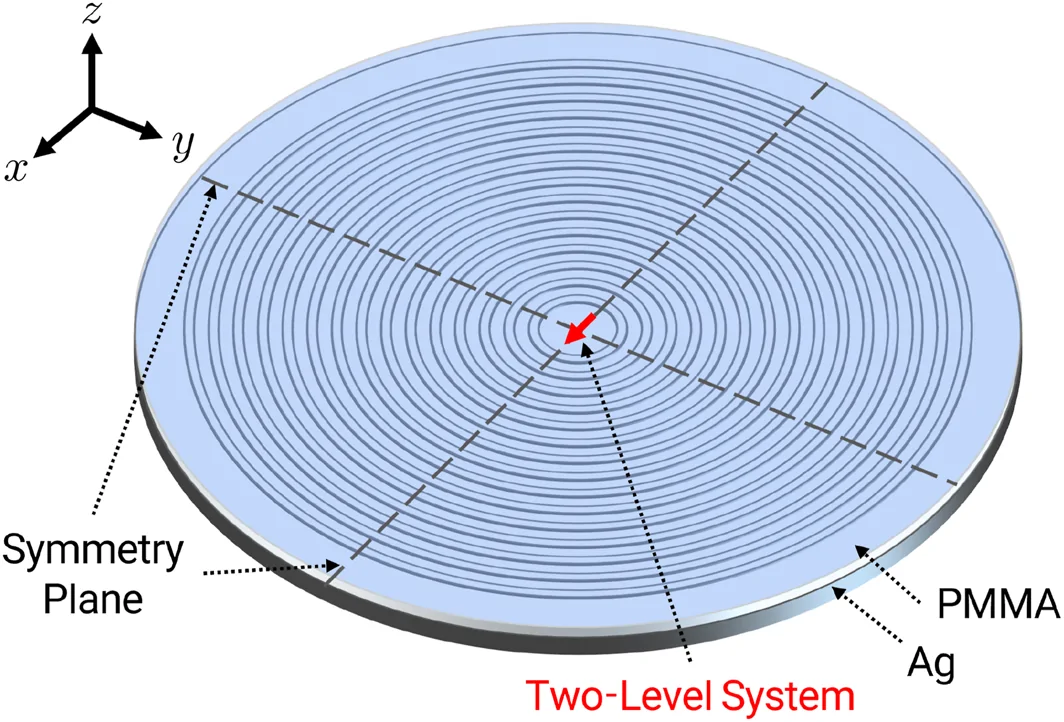
Simulation configuration based on a bull's‐eye nanoantenna structure designed to observe the Purcell effect [14]. Owing to the fourfold symmetry of the structure with respect to the TLS at its center, only one quarter of the computational domain was analyzed using one PEC symmetry plane and one PMC symmetry plane. The full‐field distribution was then reconstructed by applying the corresponding symmetry operations to the simulated quarter‐domain result.

In the SFA, the spontaneous emission rate of a two‐level emitter with transition frequency $\omega_{a}$ and dipole moment $d=\hat{x}d$ is determined by the imaginary part of the dyadic Green's function at the emitter's position $r_{a}$:

(58) Γ(SFA)=2ωa2ℏc2ϵ0d⋅ImG¯ra,ra;ωa⋅d∗=2ωa2d2ℏc2ϵ0ImGx,xra,ra;ωa.

In the three‐dimensional geometry considered in this work, the Purcell factor is directly related to the local Green's function via

(59) FP=Γ(SFA)Γ0=3λ×ImGx,xra,ra;ωa.

In addition, the instantaneous decay rate was extracted from the population dynamics as $\Gamma_{\text{inst}}\left(t\right)=-d\;\log|C\left(t\right){|}^{2}/dt$, and the corresponding instantaneous Purcell factor, $F_{p,\text{inst}}\left(t\right)=\Gamma_{\text{inst}}\left(t\right)/\Gamma_{0}$, was compared with the SFA prediction.

Figure 6 compares the non‐Markovian emitter dynamics obtained from Equation (45) with the Markovian population decay constructed using the SFA decay rate in Equation (58). The left panel shows the time evolution of the excited‐state population. Its departure from the corresponding Markovian exponential decay demonstrates the influence of environmental memory and retardation on the emitter dynamics.

**FIGURE 6:**
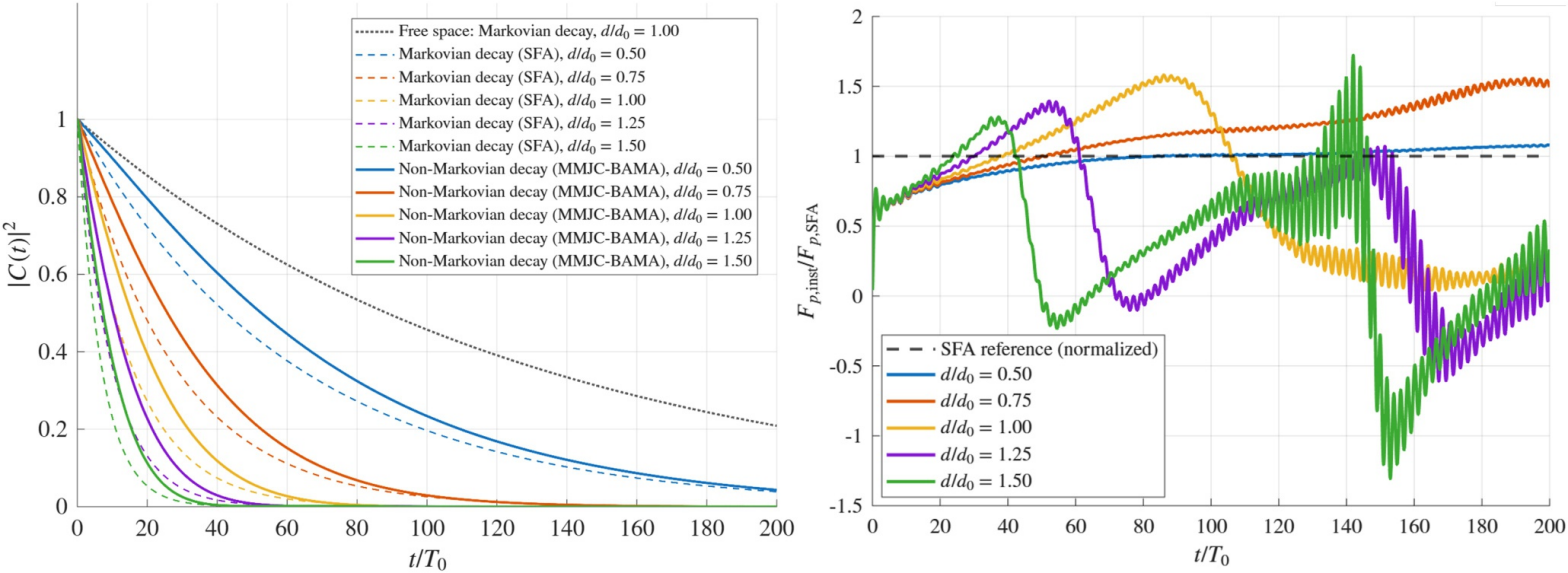
Comparison of the SFA and MMJC–BAMA results for different dipole strengths at an atomic transition wavelength of $\lambda_{a}=650$ nm. Left: time evolution of the atomic population for different normalized dipole strengths $d/d_{0}$, where $d_{0}$ denotes the characteristic dipole scale of the system. Right: comparison between the instantaneous decay rate extracted from the MMJC–BAMA population and the normalized SFA result. Here, $T_{0}=2\pi /\omega_{a}$ denotes the TLS transition period.

The right panel presents the instantaneous decay rate extracted from the non‐Markovian population dynamics. At early times, the instantaneous decay rate is close to zero and subsequently exhibits both increases and decreases as the system dynamics evolve. For smaller dipole moments, the time‐dependent instantaneous decay rate remains broadly consistent with the SFA prediction. As the dipole moment increases, however, the deviations from the SFA‐predicted rate become increasingly pronounced. For larger dipole moments, the instantaneous decay rate even becomes negative over certain time intervals, indicating a temporary recovery of the excited‐state population and population backflow characteristic of non‐Markovian dynamics.

In addition to the TLS population dynamics, the spatial distribution of the single‐photon detection probability, $|E_{\text{spa}}\left(r,t\right){|}^{2}$, was evaluated using Equation (57). The resulting distributions are shown in Figure 7. At early times, the emitted single‐photon field remains localized near the TLS. As the system dynamics evolve, the field emitted from the TLS interacts with the surrounding nanostructure and develops a directional radiation pattern. At later times, the overall radiation intensity decreases as the emission dynamics decay. Because the simulation is performed in three dimensions, the distinct spatial distributions of the emitted field can also be resolved in the xz and yz cross sections. These results show that the present methodology successfully tracks the relevant field degrees of freedom and enables the direct reconstruction of the spatiotemporal distribution of the single‐photon amplitude.

**FIGURE 7:**
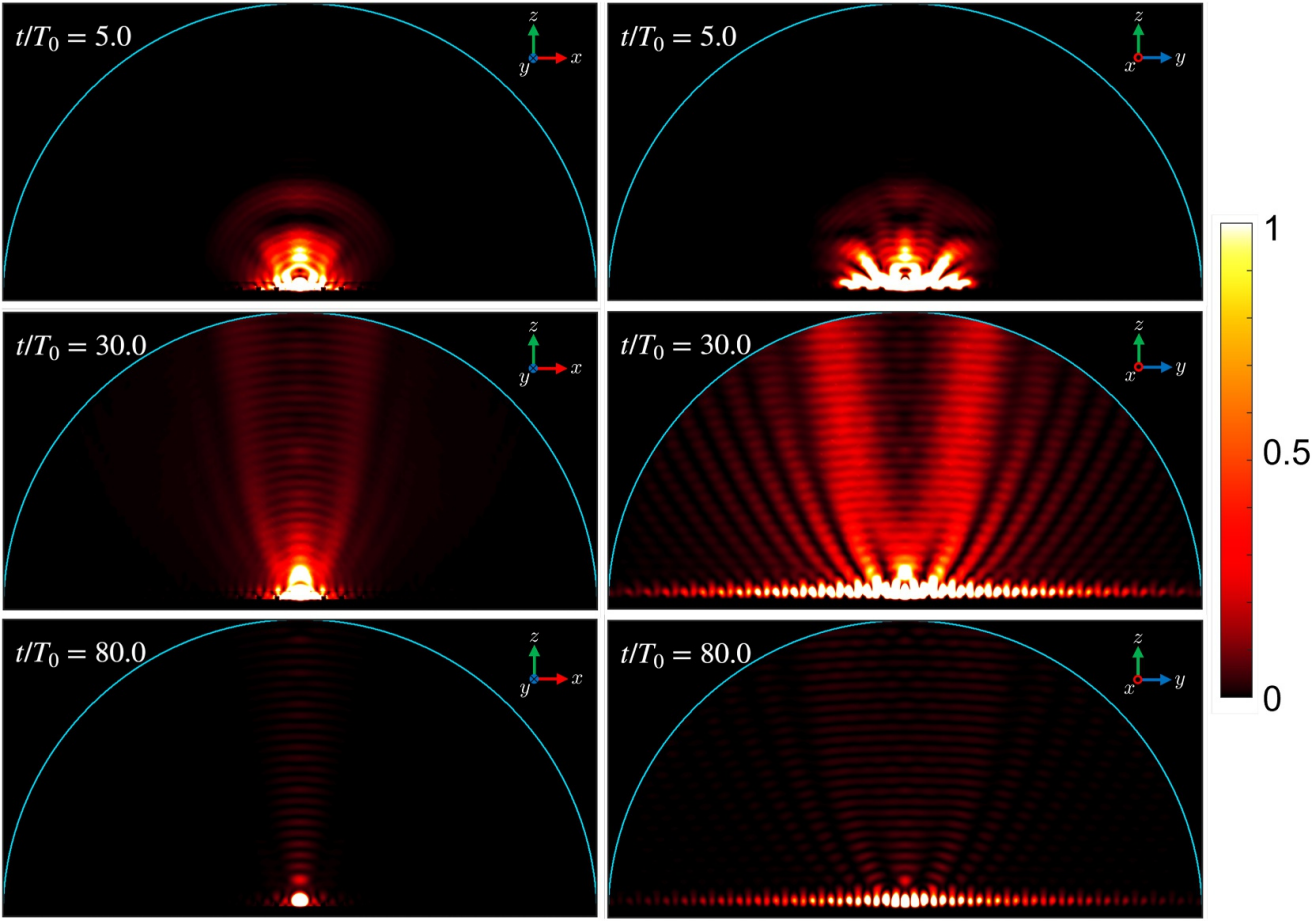
Spatial distributions of the single‐photon detection probability $|E_{\text{spa}}{|}^{2}$ at $t/T_{0}=5,\;30,\;80$ for the bull's‐eye nanoantenna. The left and right columns show the distributions in the xz and yz cross sections, respectively. The color scale represents the relative single‐photon detection probability in arbitrary units.

## 5 Conclusion and Future Works

In this work, we developed a computational framework based on the M–LN formalism for modeling quantum–plasmonic phenomena. Within the Heisenberg picture, we demonstrated HOM‐type two‐photon interference in a plasmonic beam‐splitting platform by expanding the electric‐field operator using BA–MA modes and modeling incident single‐photon states through BA‐field superpositions. A key result is that the second‐order correlation and single‐photon detection probability can be evaluated using the derived quantum–optical expressions, with the required field envelopes reconstructed from the classical electromagnetic simulations. Within the Schrödinger picture, we proposed a MMJC formulation that explicitly incorporates BA–MA coupling while avoiding the computational intractability of sampling the full continuum‐mode degeneracy space. By lumping out the BA–MA degeneracy degrees of freedom and defining frequency‐dependent effective variables, our approach captures atom–plasmon–radiation coupling without explicitly propagating the full degenerate continuum. Using this method, we analyzed single‐photon emission from a two‐level system embedded in a bull's‐eye plasmonic antenna and examined how the antenna geometry influences the emitted field distribution. Together, these results demonstrate the applicability of the M–LN formalism to the two quantum–plasmonic problems considered in this work.

The present framework admits several natural extensions. First, it can be generalized to multi‐emitter configurations, enabling the analysis of cooperative quantum effects arising from atomic inhomogeneous broadening and dipole–dipole interactions mediated by reactive near fields. Such extensions would allow systematic investigation of superradiant and subradiant responses, entanglement dynamics, and collective non‐Markovian behavior in plasmonic environments containing multiple quantum emitters. Second, the BA–MA modal quantization provides a suitable foundation for modeling open and dissipative plasmonic systems in the ultra‐strong and deep‐strong coupling regimes. By incorporating the minimal‐coupling Hamiltonian, including its nonlinear contributions, and combining it with numerical techniques such as matrix–product–state representations, the framework may be applied to regimes beyond the rotating‐wave and Markov approximations. This offers a controlled theoretical setting for studying nonperturbative light–matter interactions in quantum plasmonics. Finally, the proposed approach can be adapted to photonic integrated circuit platforms incorporating plasmonic cavity quantum electrodynamics elements. Because the BA–MA formalism is compatible with large‐scale computational electromagnetic solvers, it is well suited for modeling on‐chip plasmonic resonators, waveguides, and hybrid plasmonic–dielectric structures in realistic open and lossy environments. Such extensions would facilitate quantitative device‐level analysis of integrated quantum photonic systems.

## Author Contributions

**Jisang Seo:** investigation, validation, software, data curation, visualization, writing – original draft, writing – review and editing. **Hyunwoo Choi:** investigation. **Thomas E. Roth:** writing – review and editing, investigation, funding acquisition. **Jie Zhu:** investigation. **Weng Cho Chew:** supervision, funding acquisition. **Dong‐Yeop Na:** conceptualization, methodology, software, data curation, investigation, validation, formal analysis, supervision, funding acquisition, visualization, project administration, resources, writing–original draft, writing – review and editing.

## Conflicts of Interest

The authors declare no conflicts of interest.

## Data Availability

The data that support the findings of this study are available from the corresponding author upon reasonable request.
